# Azimuth Phase Center Adaptive Adjustment upon Reception for High-Resolution Wide-Swath Imaging

**DOI:** 10.3390/s19194277

**Published:** 2019-10-02

**Authors:** Wei Xu, Jialuo Hu, Pingping Huang, Weixian Tan, Yifan Dong

**Affiliations:** 1 College of Information Engineering, Inner Mongolia University of Technology, Hohhot 010051, China; hujialuo166@163.com (J.H.); wxtan@imut.edu.cn (W.T.); yfdong@imut.edu.cn (Y.D.); 2 Inner Mongolia Key Laboratory of Radar Technology and Application, Hohhot 010051, China

**Keywords:** synthetic aperture radar (SAR), high resolution wide swath (HRWS), azimuth multichannel reconstruction, phase center adaptation, false targets suppression

## Abstract

A spaceborne azimuth multichannel synthetic aperture radar (SAR) system can effectively realize high resolution wide swath (HRWS) imaging. However, the performance of this system is restricted by its two inherent defects. Firstly, non-uniform sampling is generated if the pulse repetition frequency (PRF) deviates from the optimum value. Secondly, multichannel systems are very sensitive to channel errors, which are difficult to completely eliminate. In this paper, we propose a novel receive antenna architecture with an azimuth phase center adaptive adjustment which adjusts the phase center position of each sub-aperture to improve multichannel SAR system performance. On one hand, the optimum value of the PRF can be adaptively adjusted within a certain range by adjusting receiving phase centers to obtain uniform azimuth sampling. On the other hand, false targets introduced by residual channel errors after azimuth multichannel error compensation can be further suppressed. The effectiveness of the proposed method to compensate for non-uniform sampling and suppress false targets is verified by simulation experiments.

## 1. Introduction

Synthetic aperture radar (SAR) is an extremely important device for the application of earth observation, especially where there is cloud or poor atmosphere conditions [1,2,3]. It is also widely used in military surveillance and civilian remote sensing [4,5,6,7]. The azimuth multichannel spaceborne SAR systems, usually working with a single transmit antenna and multiple received sub-apertures, can effectively overcome the contradiction between azimuth resolution and range mapping swath width [8,9,10]. However, in practical multichannel high resolution wide swath (HRWS) SAR systems, there are two problems which affect SAR system performance. Firstly, azimuth non-uniform sampling is generated if the pulse repetition frequency (PRF) is not selected as the optimum value [11,12,13]. Secondly, the multichannel system is very sensitive to channel errors, which are difficult to completely eliminate. 

Many researchers have done a lot of research on the two mentioned issues, but some problems still exist. Firstly, azimuth non-uniform sampling in HRWS SAR can be overcome by the recent proposed azimuth multichannel reconstruction algorithms, but these are only prepared for azimuth band-limited signals [11,14,15,16]. However, the azimuth echo signal received by a practical SAR system is non-band-limited, and false targets still exist after reconstruction. To solve this problem, a novel transmit antenna architecture which allows for the adjustment of the transmit phase center position through the activation of a specific number of elements on the corresponding location of the transmit antenna was proposed in [17]. However, a decreased number of activated elements reduces the transmit antenna gain and the transmit signal power. Secondly, azimuth channel errors can be compensated for by many recently proposed compensation methods [18,19,20]. However, since factors such as the manufacturing process, temperature and radiation that affect the characteristics of antenna are not fixed, these methods are unable to perfectly estimate or compensate for multichannel errors, especially when the signal-to-noise ratio (SNR) of the obtained raw data is not high enough. The residual channel errors still cause high false targets which cannot be ignored and need to be further suppressed.

In this paper, a novel receiving antenna architecture that allows for the adjustment of the receiving phase center position of each sub-aperture through the closing of the corresponding elements on the side of each sub-antenna is proposed. In this approach, the transmitted signal power is not reduced, but the reduced receive antenna gain is compensated for by a narrow transmit antenna beam with a high antenna gain. Compared with traditional multichannel SAR systems, the novel proposed approach brings two benefits. Firstly, the optimum value of the PRF can be adaptively adjusted within a certain range by adjusting the phase center spacing of the sub-apertures. Uniform samples can be obtained if the PRF is taken in that range. Secondly, false targets caused by the residual channel errors could be further suppressed by adaptively adjusting phase center upon reception. The novel antenna structure is an improvement on the widely used conventionally phased array antenna. This improvement will hardly increase costs.

Based on the ideas above, this paper is structured as follows. Section 2 analyzes the influence of azimuthal non-uniform sampling and channel imbalance on azimuth multichannel SAR imaging. In Section 3, the basic principle of the proposed azimuth phase center adaptive adjustment upon reception is presented, and its effects on SAR system performance improvement and false targets suppression are analyzed. Simulation experiments are carried out to validate the proposed method in Section 4. Finally, this paper is concluded in Section 5. 

## 2. Influence of Azimuthal Non-Uniform Sampling and Channel Imbalance

### 2.1. Influence of Azimuthal Non-Uniform Sampling

For simplicity, only the azimuth signal of multichannel SAR is analyzed in this paper. Assuming that the number of azimuth receiving channels is an odd *N* and the slant range from the radar to the target is $R_{0}$ and the intermediate channel is used as the reference channel, the received azimuth echo signal of receiving channel *i* can be written as

(1) $$s_{i}\left(t\right)=exp\left[-j\frac{2\pi }{\lambda }\left(\sqrt{R_{0}^{2}+{\left(v_{s}t\right)}^{2}}+\sqrt{R_{0}^{2}+{\left(v_{s}t-\Delta x_{i}\right)}^{2}}\right)\right]$$

where $\Delta x_{i}$ is the phase center position of channel *i*, $\Delta x_{i}=\left(\frac{\left(N+1\right)}{2}-i\right)\cdot d_{az}$, i=1,2,⋯,N, $d_{az}$ is the phase center spacing of receiving channel, *λ* is the wavelength of the carrier, $v_{s}$ is the velocity of SAR platform, and *t* is the azimuth time.

**Figure 1:**
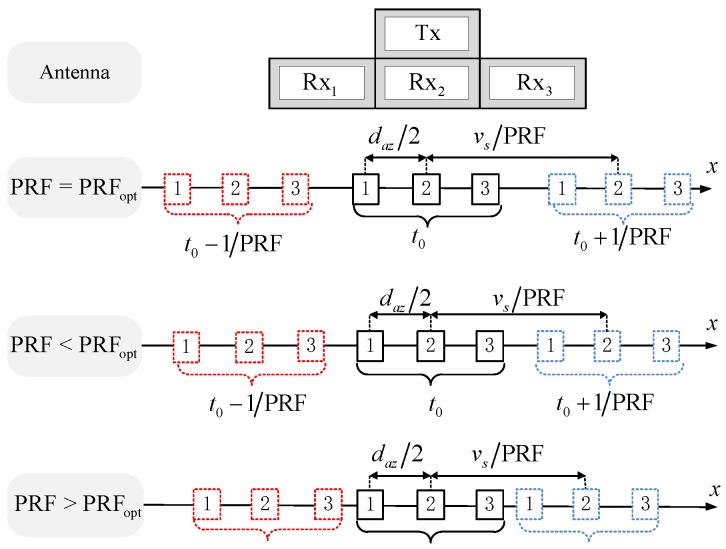
Reasons of uniform and non-uniform sampling.

Figure 1 illustrates the principle of multichannel system sampling. The equivalent sampling position can be approximately regarded as the midpoint of the transmitting position and the receiving position. In order to uniformly distribute the equivalent single-channel sampling centers, the PRF must meet Equation (2).

(2) $${\mathrm{PRF}}_{\mathrm{opt}}=\frac{2\cdot v_{s}}{N\cdot d_{az}}$$

If (2) is violated, azimuth non-uniform sampling will be induced, and false targets in azimuth will be generated [21]. 

The multichannel reconstruction algorithm introduced in [12] can effectively compensate for the non-uniform sampling in azimuth. However, for strong deviations from the optimum PRF in Equation (2), the inverse character of such an algorithm might result in a degraded system performance.

The multichannel SAR system performance of such a system with azimuth non-uniform sampling is based on multichannel reconstruction algorithms [22], and the ratio of the input to output SNR, normalized to the ratio obtained for uniform sampling is expressed as Equation (3) [12]:

(3) $$\Phi_{\mathrm{bf}}\left(\mathrm{PRF}\right)=\frac{\left(\frac{{\mathrm{SNR}}_{\mathrm{in}}}{{\mathrm{SNR}}_{\mathrm{out}}}\right)}{{\left(\frac{{\mathrm{SNR}}_{\mathrm{in}}}{{\mathrm{SNR}}_{\mathrm{out}}}\right)|}_{{\mathrm{PRF}}_{\mathrm{opt}}}}=N\cdot \sum_{j=1}^{N}E\left[{\left|P_{j}\left(f,\mathrm{PRF}\right)\right|}^{2}\right],$$

where E[⋅] represents the calculation of the mean value, and $P_{j}\left(f,\mathrm{PRF}\right)$ is the filter function of channel j. 

Assume that the reconstruction filter matrix is P(f), which is obtained by inverting matrix H(f) according to [14]. The azimuth ambiguity-to-signal ratio (AASR) multi-channel system can be written as Equation (4) [12]:

(4) $$AASR_{m}=\frac{\int_{-\frac{B_{d}}{2}}^{\frac{B_{d}}{2}}{\left|2\cdot \sum_{k=1}^{\infty }\sum_{m=m_{0}}^{N}\sum_{j=1}^{N}U_{jk}\left(f\right)\cdot P_{jm}\left(f\right)\right|}^{2}df}{\int_{-\frac{B_{d}}{2}}^{\frac{B_{d}}{2}}{\left|U\left(f\right)\right|}^{2}df}$$

with

(5) $$U_{jk}\left(f\right)=U\left(f\right)\cdot H_{jk}\left(f\right)$$

(6) $$m_{0}=max\left\{N-k+1,1\right\},$$

where $B_{d}$ is the Doppler bandwidth, U(f) is the spectrum of the equivalent monostatic SAR signal, $P_{j}\left(f\right)$ is the reconstruction filter of channel j, $P_{jm}\left(f\right)$ is *m*-th filter of $P_{j}\left(f\right)$, $H_{j}\left(f\right)$ is the pre-filter of channel *j*, and $H_{jk}\left(f\right)$ is the *k*-th filter in $H_{j}\left(f\right)$. 

According to (3) and (4), both the SNR scaling factor and the AASR are related to the reconstruction matrix P(f). A PRF deviating strongly from the optimum value will lead to an irreversible H(f) or very different eigenvalues of H(f). As a result, the multichannel SAR system performance would be significantly declined.

**Figure 2:**
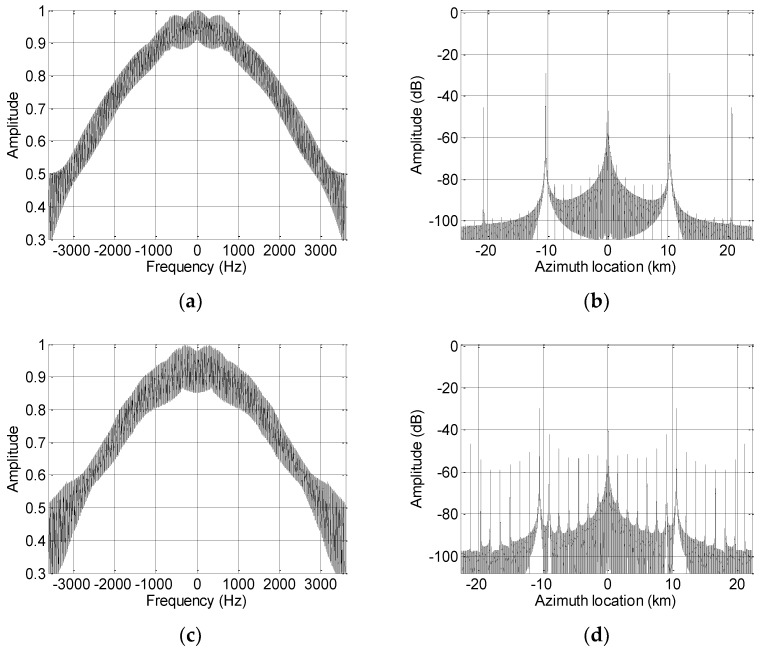
Influence of the pulse repetition frequency (PRF) deviating from the optimum value on imaging performance. (**a**) Reconstructed spectrum with the optimum PRF; (**b**) reconstructed pulse compression result with the optimum PRF; (**c**) reconstructed spectrum with the non-optimum PRF; and (**d**) reconstructed pulse compression result with the non-optimum PRF.

Using the simulation parameters listed in Table 1, Figure 2 shows that the reconstruction filter causes a degraded imaging performance. Figure 2a,b show the spectrum and pulse compression, respectively, result of the equivalent single-channel signal when the PRF is the optimum value. Since the azimuth SAR echo signal is non-band-limited, several false targets caused by azimuth ambiguity appear in the compression result. When the actual PRF of the system takes a non-optimum value, the reconstruction result of the non-band-limited signal is shown in Figure 2c,d. It can be seen that the tiny false targets in Figure 2b are significantly enlarged in Figure 2d by the non-optimum PRF. 

**Figure 3:**
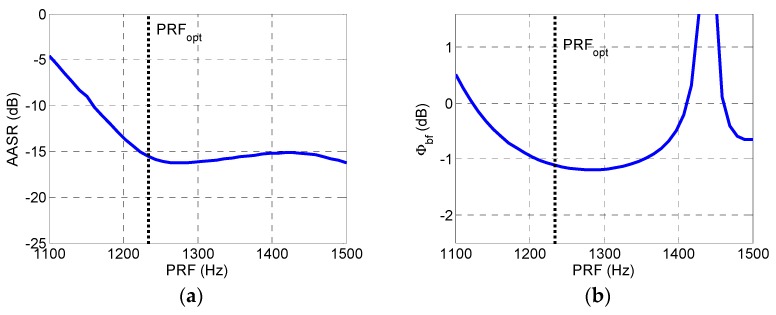
Simulated ambiguity-to-signal ratio (AASR) and signal-to-noise ratio (SNR) scaling factor of conventional reconstruction approach. (**a**) Simulated AASR of conventional reconstruction approach and (**b**) simulated SNR scaling factor $\Phi_{\mathrm{bf}}$ of conventional reconstruction approach.

Assuming that the desired PRF range of the system is 1100–1500 Hz and the optimum PRF is 1234.3 Hz according to Table 1, the resulting azimuth ambiguity-to-signal ratio (*AASR_N_*) and SNR scaling factor ($\Phi_{\mathrm{bf}}$) are shown in Figure 3. With the growth of the PRF, the AASR continues to decline when the PRF is lower than the optimum value of 1234.3 Hz, but the AASR rises when the PRF exceeds the ideal value, as is shown in Figure 3a. From Figure 3b, it can be seen that sufficiently low values of $\Phi_{\mathrm{bf}}$ are shown from 1100 up to 1370 Hz, but unacceptably high values are generated for the PRF range above 1400 Hz. The phase center adjustment method proposed in this article can obviously decrease the AASR and significantly suppress the unacceptably high values of $\Phi_{\mathrm{bf}}$.

### 2.2. Influence of Channel Imbalance

In actual multi-channel SAR systems, there are always channel errors among channels. These errors cause false targets that seriously reduce the quality of imaging. Assuming that the phase error of the *n*-th channel is $\phi_{n}$ and the amplitude error is $a_{n}$, the echo signal model of channel *n* can be expressed as follows:

(7) $$s_{n}\left(t\right)=a_{n}exp\left(j\phi_{n}\right)exp\left[-j\frac{2\pi }{\lambda }\left(\sqrt{R_{0}^{2}+{\left(v_{s}t\right)}^{2}}+\sqrt{R_{0}^{2}+{\left(v_{s}t-\Delta x_{n}\right)}^{2}}\right)\right].$$

In the process of multi-channel signal combination, *N* − 1 zeros need to be added between each sampling point of each channel, and the equivalent single channel signal can be written as:

(8) $$s\left(m\right)=\sum_{n=0}^{N-1}s_{n}^{0}\left(m\right)\;,$$

where *m* represents integers associated with sampling time and $s_{n}^{0}\left(m\right)$ represents signals after adding zeros of channel *n*.

According to (8), the discrete time Fourier transform (DTFT) [23] of $s_{n}^{0}\left(m\right)$ is:

(9) $$S_{n}^{0}\left(e^{j\omega }\right)=\frac{1}{N}\sum_{k=0}^{N-1}e^{-j2\pi \frac{nk}{N}}S\left(e^{j\left(\omega -\frac{2\pi k}{N}\right)}\right),$$

where *k* represents integers associated with sampling frequency. Denote the digital frequency of (9) using analog frequency, and the spectrum of signals after adding zeros of channel *n* can be derived as:

(10) $$\begin{array}{c} S_{n}^{0}\left(f\right)=a_{n}exp\left(j\phi_{n}\right)rect\left(\frac{f}{B_{d}}\right)exp\left(-j\pi \frac{f^{2}}{K_{a}}\right)\\ +a_{n}exp\left(j\phi_{n}\right)\sum_{l=0}^{1}\sum_{k=1}^{N-1}W\left(f\right)exp\left(\frac{-j2\pi nk}{N}\right)exp\left(-j\pi \frac{{\left(f+l\cdot \mathrm{PRF}-\frac{k\cdot \mathrm{PRF}}{N}\right)}^{2}}{K_{a}}\right), \end{array}$$

where $K_{a}$ is the azimuth frequency modulation (FM) rate, and *f* is the frequency variable in Hz. W(f) is given by:

(11) $$W\left(f\right)=\mathrm{rect}\left[\frac{f-\frac{\left(\frac{k\cdot \mathrm{PRF}}{N}-l\cdot \mathrm{PRF}\right)}{2}-{\left(-1\right)}^{l}\;\frac{\left(\mathrm{PRF}-B_{d}\right)}{4}}{{\left(-1\right)}^{l+1}\left(\frac{k\cdot \mathrm{PRF}}{N}-l\cdot \mathrm{PRF}\right)+\frac{\left(B_{d}+\mathrm{PRF}\right)}{2}}\right].$$

Due to the spectrum components corresponding to other *l* values move to the right and fall out, the value of *l* is only 0 and 1. 

According to (8) and (10), the pulse compression result of the combined signal can be derived as:

(12) $$\begin{array}{c} s_{out}\left(t\right)=B_{d}\sin c\left(B_{d}t\right)\sum_{n=0}^{N-1}a_{n}exp\left(j\phi_{n}\right)+\sum_{l=0}^{1}\sum_{k=1}^{N-1}f_{l,k}\left(t\right)\cdot \\ \sum_{n=0}^{N-1}exp\left(\frac{-j2\pi nk}{N}\right)a_{n}exp\left(j\phi_{n}\right), \end{array}$$

where $f_{l,k}\left(t\right)$ is a function introduced to simplify the written, and it has nothing to do with amplitude and phase errors. According to (12), there are 2(N−1) false targets, and the position of each false target is shown as follows:

(13) $${\mathrm{POS}}_{l,k}=\frac{\left(l\cdot \mu -\frac{k\cdot \mu }{N}\right)}{T_{a}}\;,$$

where l=0,1, k=1,2,…,N−1, *μ* is the oversampling rate, and $T_{a}$ is the synthetic aperture interval. Assuming that the channel characteristics are independent of frequency, the false target-to-peak ratio of the false target at ${\mathrm{POS}}_{l,k}$ is as follows:

(14) $${\mathrm{PGR}}_{l,k}=20{log}_{10}\left(\frac{\left|\sum_{n=0}^{N-1}a_{n}exp\left(j\phi_{n}\right)exp\left(\frac{-j2\pi nk}{N}\right)\right|}{\left|\sum_{n=0}^{N-1}a_{n}exp\left(j\phi_{n}\right)\right|}\right)+20{log}_{10}\left(\frac{g_{l,k}}{B_{d}}\right),$$

in which $g_{l,k}$ is given by $g_{l,k}={\left(-1\right)}^{l+1}\left(\frac{k\cdot \mathrm{PRF}}{N}-l\cdot \mathrm{PRF}\right)+\frac{\left(B_{d}+\mathrm{PRF}\right)}{2}$ and is independent with amplitude and phase errors.

**Figure 4:**
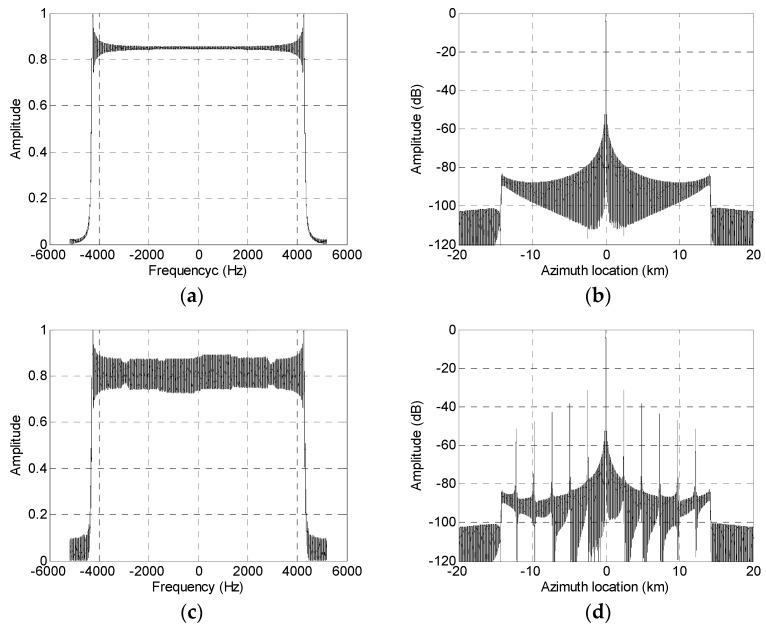
Influence of channel imbalance. (**a**) Reconstructed spectrum with no channel errors; (**b**) reconstructed pulse compression result with no channel errors; (**c**) reconstructed spectrum with channel errors; and (**d**) reconstructed pulse compression result with no channel errors.

With parameters listed in Table 2, a simulation experiment to reconstruct the echo signal with channel errors is shown in Figure 4. A reconstructed spectrum and compression result of the system with no channel errors is shown in Figure 4a,b. Figure 4c,d show the reconstruction results in the presence of amplitude errors within 0.5 dB and phase errors of 1–5 degrees. It can be seen that these tiny channel errors that may be residual after channel error compensation can cause false targets of around −35 dB. False targets of such intensity still cause a reduction in image quality and should be further suppressed.

## 3. Phase Center Adjustment upon Reception

**Figure 5:**
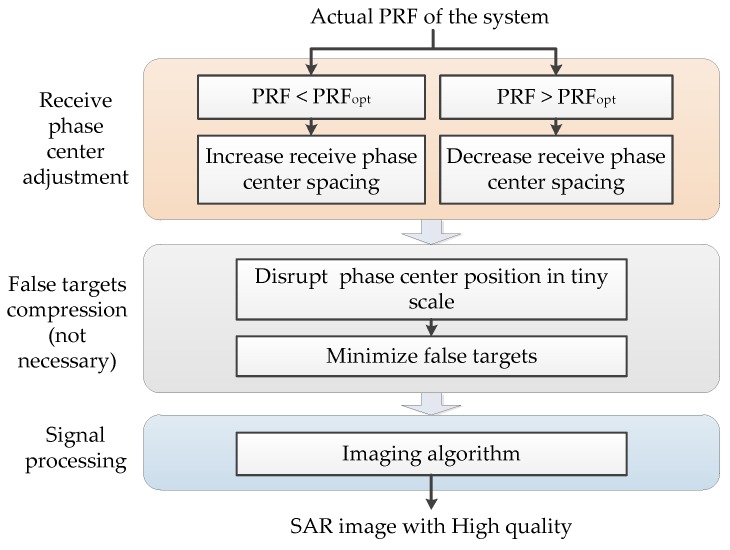
The working process of the innovative system.

This section introduces an innovative receive antenna architecture in azimuth which allows for the compensation of the non-optimum PRF values and the suppression of false targets by phase center adjustment upon reception. The working process of the innovative system is shown in Figure 5.

### 3.1. System Architecture and Basic Principle

**Figure 6:**
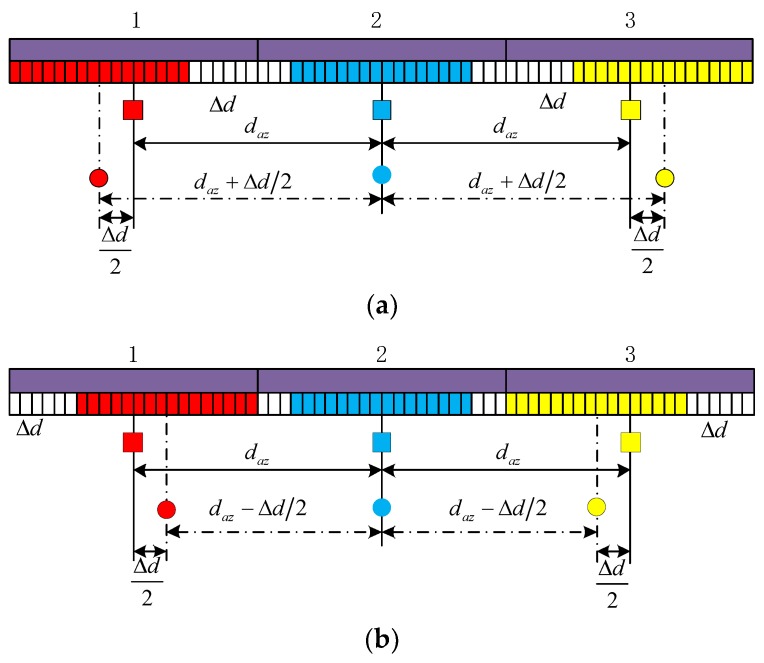
System architecture and basic principle. (**a**) Adjustment to increase phase center spacing and (**b**) adjustment to reduce phase center spacing.

Compared with traditional multi-channel SAR systems, such as systems used by satellites RADARSAT-2 (Canada, 2007), TerraSAR-X (Germany, 2007) and Sentinel-1A (launched by European Space Agency, in 2014), each receiving sub-antenna of the innovative system consists of a large number of individually controllable elements. Such an aperture permits the changing of the position and the length of the effective receiving sub-apertures on the antenna by activating respective elements. The phase center position of the receiving sub-apertures can be adaptively adjusted by turning off a part of elements on the antenna. As an example, a system with three receive apertures is shown in Figure 6.

Closing a part of elements inside a sub-aperture can move its phase center outward and increase the phase center spacing, as shown in Figure 6a, while the inward movement of the phase center can be achieved by closing the elements on the outside of the sub-aperture, as shown in Figure 6b. To ensure the effective length of each antenna is equal, the same number of elements on each aperture should be turned off. The phase center position can be adjusted by controlling the amount of closed elements on each side of the aperture. After adjustment, the phase center spacing can be uniform or non-uniform, which should be decided according to practical needs. 

### 3.2. Effect of Receiving Sub-Aperture Phase Center Adjustment on Azimuth Non-Uniform Sampling

Take the case where the phase center spacing is reduced as an example. If the length of the Δd antenna on left side of channel 1 and the right side of channel *N* are invalid, the phase center of the two sub-apertures moves inward by $\frac{\Delta d}{2}$ and the distance between the phase centers of channel 1 and channel *N* is reduced by Δd. In the case where the phase center is evenly distributed, the new phase center spacing is:

(15) $$d_{\mathrm{az}\_\mathrm{new}}=\frac{\left(N-1\right)\cdot d_{\mathrm{az}}-\Delta d}{N-1}=d_{\mathrm{az}}-\frac{\Delta d}{N-1}.$$

To ensure that the phase centers are uniformly distributed, the phase center of channel *n* should move inward by:

(16) $$x_{n}=\left|\frac{N+1}{2}-n\right|\cdot \frac{\Delta d}{N-1},n=1,2,\ldots ,N.$$

The length of the invalid antenna on the outer side of channel n should be $2x_{n}$ longer than the invalid antenna on the inner side:

(17) $$\Delta d_{n,\mathrm{outer}}-\Delta d_{n,\mathrm{inner}}=2x_{n}=\frac{2\Delta d}{N-1}\cdot \left|\frac{N+1}{2}-n\right|.$$

Since the number of closed elements in each channel must be equal (assumed to be Δd), the length of the invalid part on both sides of channel *n* should satisfy:

(18) $$\Delta d_{n,\mathrm{outer}}+\Delta d_{n,\mathrm{inner}}=\Delta d.$$

Combine Formulas (17) and (18) together, and then $\Delta d_{n,\mathrm{outer}}$ and $\Delta d_{n,\mathrm{inner}}$ can be written as:

(19) $$\Delta d_{n,\mathrm{outer}}=\frac{\Delta d}{2}+\frac{\Delta d}{N-1}\cdot \left|\frac{N+1}{2}-n\right|$$

(20) $$\Delta d_{n,\mathrm{inner}}=\frac{\Delta d}{2}-\frac{\Delta d}{N-1}\cdot \left|\frac{N+1}{2}-n\right|\;.$$

Because of the size of individually controllable antenna elements, the receive center of antenna in practical system cannot be adjusted arbitrarily but can only be selected in a series of discrete positions. Assuming that each receive antenna consists of a number of K elements, the number of closed elements on each antenna can be written as:

(21) $$p=\mathrm{round}\left\{\Delta d\cdot \frac{K}{l_{a}}\right\},$$

in which the operator round {⋅} indicates the calculation of rounding integers. The number of invalid elements on the outer side and the inner side of the antenna can be written as:

(22) $$p_{n,\mathrm{outter}}=\mathrm{round}\left\{\left[\frac{\Delta d}{2}+\frac{\Delta d}{N-1}\cdot \left|\frac{N+1}{2}-n\right|\right]\cdot \frac{K}{l_{a}}\right\}\;.$$

(23) $$p_{n,\mathrm{inner}}=\mathrm{round}\left\{\left[\frac{\Delta d}{2}-\frac{\Delta d}{N-1}\cdot \left|\frac{N+1}{2}-n\right|\right]\cdot \frac{K}{l_{a}}\right\}$$

If the actual value of the PRF is ${\mathrm{PRF}}_{\mathrm{act}}$, which is higher than the optimum value, the phase center spacing should be reduced by $\Delta d_{az}$, which is given by:

(24) $$\Delta d_{az}=d_{az}-\frac{2v_{s}}{N\cdot {\mathrm{PRF}}_{\mathrm{act}}}.$$

According to (15), the length of closed elements on each receive antenna should be:

(25) $$\Delta d=\left(d_{az}-\frac{2v_{s}}{N\cdot {\mathrm{PRF}}_{\mathrm{act}}}\right)\cdot \left(N-1\right).$$

The number of closed elements on each antenna can be derived as:

(26) $$p=\mathrm{round}\left\{\frac{K}{l_{a}}\cdot \left(N-1\right)\cdot \left(d_{az}-\frac{2v_{s}}{N\cdot {\mathrm{PRF}}_{\mathrm{act}}}\right)\right\}\;.$$

The respective number of closed elements on the outer side and inner side on channel n is given by:

(27) $$p_{n,\mathrm{outter}}=\mathrm{round}\left\{\left[\left(d_{az}-\frac{2v_{s}}{N\cdot {\mathrm{PRF}}_{\mathrm{act}}}\right)\cdot \frac{\left(N-1\right)}{2}+\left(d_{az}-\frac{2v_{s}}{N\cdot {\mathrm{PRF}}_{\mathrm{act}}}\right)\cdot \left|\frac{N+1}{2}-n\right|\right]\cdot \frac{K}{l_{a}}\right\}$$

(28) $$p_{n,\mathrm{inner}}=\mathrm{round}\left\{\left[\left(d_{az}-\frac{2v_{s}}{N\cdot {\mathrm{PRF}}_{\mathrm{act}}}\right)\cdot \frac{\left(N-1\right)}{2}-\left(d_{az}-\frac{2v_{s}}{N\cdot {\mathrm{PRF}}_{\mathrm{act}}}\right)\cdot \left|\frac{N+1}{2}-n\right|\right]\cdot \frac{K}{l_{a}}\right\}.$$

Using the method discussed above, if the number of closed elements on each channel is given by p, the optimum PRF of the system can be written as:

(29) $${\mathrm{PRF}}_{\mathrm{opt}}=\frac{2v_{s}}{N\cdot \left(d_{az}\pm \frac{p\cdot l_{a}}{K\cdot \left(N-1\right)}\right)}.$$

The “±” indicates the increase or decrease of the phase center spacing. In practice, in order to ensure the receiving performance of the antenna, a limited number of elements can be turned off. If the maximum ratio of the closed elements number to the total number on each antenna is η, the number of closed elements can be written as:

(30) $$p_{m}=\mathrm{round}\left\{\eta \cdot K\right\}$$

The range of the adjusted optimum PRF is:

(31) $$\frac{2v_{s}}{N\cdot \left(d_{az}+\frac{p_{m}\cdot l_{a}}{K\cdot \left(N-1\right)}\right)}\leq {\mathrm{PRF}}_{\mathrm{opt}}\leq \frac{2v_{s}}{N\cdot \left(d_{az}-\frac{p_{m}\cdot l_{a}}{K\cdot \left(N-1\right)}\right)}.$$

The minimum value of ${\mathrm{PRF}}_{\mathrm{opt}}$ is:

(32) $${\mathrm{PRF}}_{\mathrm{opt}\_\min}=\frac{2v_{s}}{N\cdot \left(d_{az}+\frac{p_{m}\cdot l_{a}}{K\cdot \left(N-1\right)}\right)};$$

and the maximum value is:

(33) $${\mathrm{PRF}}_{\mathrm{opt}\_\max}=\frac{2v_{s}}{N\cdot \left(d_{az}-\frac{p_{m}\cdot l_{a}}{K\cdot \left(N-1\right)}\right)}.$$

This is to say, if the practical PRF of the system varies between ${\mathrm{PRF}}_{\mathrm{opt}\_\min}$ and ${\mathrm{PRF}}_{\mathrm{opt}\_\max}$, a uniformly (at least approximately uniformly) sampled signal can be obtained by adjusting the phase center spacing. Consequently, the multichannel reconstruction algorithm can be omitted, and the computational complexity can be reduced compared with conventional systems. If the PRF is outside the optimum PRF range, the phase center spacing should be adjusted to the minimum or maximum value that makes the optimum PRF closest to the actual PRF of the system. In this case, although the non-uniformity of the samples is reduced, the sampling is still non-uniform, and the reconstruction algorithm is still needed. Therefore, the complexity of processing is approximately equal to that of traditional systems.

**Figure 7:**
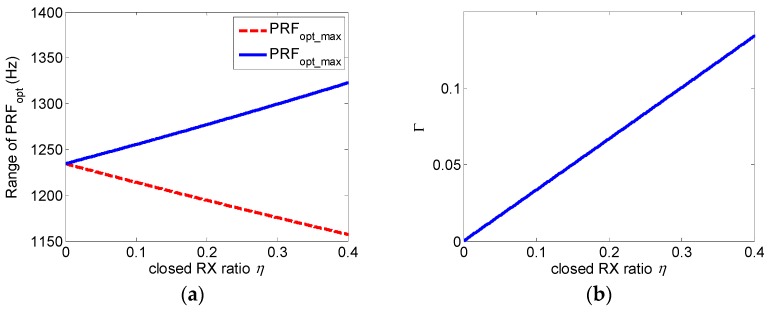
Relationship between the optimum PRF and the invalid receiving antenna ratio η. (**a**) Maximum (solid blue line) and minimum (red dotted line) value of the optimum PRF and (**b**) range expansion ratio of the optimum PRF.

The effect of the largest proportion of closed elements on the range of the optimum PRF is shown in Figure 7, where Γ is as follows:

(34) $$\Gamma =\frac{{\mathrm{PRF}}_{\mathrm{opt}\_\max}-{\mathrm{PRF}}_{\mathrm{opt}\_\min}}{{\mathrm{PRF}}_{\mathrm{opt}}}.$$

### 3.3. Suppression of False Target by Adjusting Receiving Sub-Aperture Phase Center

In Section 2.2, the expressions of the position and intensity of false targets caused by channel imbalance are analyzed. For the convenience of analysis, the false target-to-peak ratio of the false target at ${\mathrm{POS}}_{l,k}$ given by (14) can be rewritten as:

(35) $${\mathrm{PGR}}_{l,k}=20log_{10}\left(\frac{\left|\sum_{n=0}^{N-1}a_{n}exp\left(j\phi_{n}\right)exp\left(\frac{-j4\pi k\left|X_{n}\right|}{L}\right)\right|}{\left|\sum_{n=0}^{N-1}a_{n}exp\left(j\phi_{n}\right)\right|}\right)+20log_{10}\left(\frac{g_{l,k}}{B_{d}}\right),$$

where L represents the length of the antenna and $X_{n}$ represents the phase center position of channel n. If the phase centers of sub-apertures are adjusted, the antenna length *L* can be written as:

(36) $$L=L_{a}-\frac{l_{a}\cdot q_{1,l}}{K}-\frac{l_{a}\cdot q_{N,r}}{K},$$

where $L_{a}$ is the length of receive antenna before adjusting, $q_{n,l}$ is the number of inactive elements on the left side of the antenna *n*, and $q_{n,r}$ is the number of inactive elements on the right side of the antenna *n*. The value of $X_{n}$ can be written as:

(37) $$X_{n}=\left(\frac{N+1}{2}-n\right)\cdot d_{az}+\frac{l_{a}\cdot \left(q_{n,r}+q_{\frac{\left(N+1\right)}{2},l}-q_{\frac{\left(N+1\right)}{2},r}-q_{n,l}\right)}{2K}.$$

The value of $X_{n}$ can be adaptively adjusted to generate an additional phase which can cancel a part of the phase error. Based on this, the molecule of the first item in Formula (35) can be minimized by adjusting the phase center position of each channel, and the false-target-to-peak-ratio can be suppressed. The false-target-to-peak ratio can be reduced to:

(38) $${\mathrm{PGR}}_{l,k}=20log_{10}\left(\frac{\min\left\{\left|\sum_{n=0}^{N-1}a_{n}exp\left(j\phi_{n}\right)exp\left(\frac{-j4\pi k\left|X_{n}\right|}{L}\right)\right|\right\}}{\left|\sum_{n=0}^{N-1}a_{n}exp\left(j\phi_{n}\right)\right|}\right)+20log_{10}\left(\frac{g_{l,k}}{B}\right)$$

Though false targets can be suppressed by simply scrambling the uniformly distributed phase center position on a tiny scale, it is often necessary to compare several different results for optimal suppression. As a result, computational complexity and time-consumption is increased, and the increased complexity and time-consumption are related to the number of comparisons. Assuming that *n* different suppression results are compared, the computational complexity is *n* times higher than that of a traditional multichannel system.

**Figure 8:**
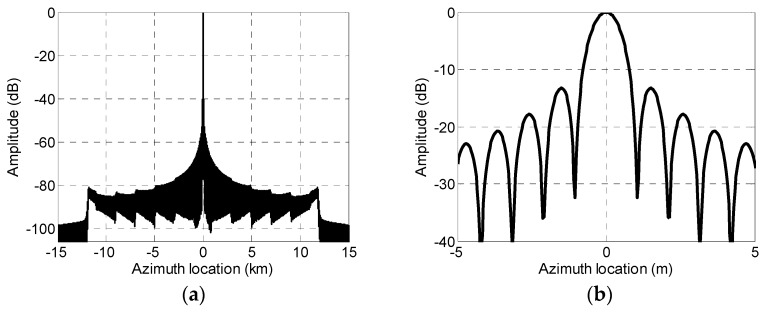
Reconstruction of non-uniform phase center echo signal without channel error. (**a**) Reconstructed pulse compression result with no channel errors and (**b**) magnified reconstructed pulse compression result with no channel errors.

The reconstruction result of error-free signals after phase center adaptive adjustment is shown in Figure 8. It can be seen that adjusting phase center non-uniformly does not affect the reconstruction results for multichannel signals.

## 4. Simulation and Performance Analysis

To validate the proposed receiving phase center adaptive adjustment approach, simulations were carried out. Simulation parameters are listed in Table 1. 

### 4.1. Effect on Azimuth Non-Uniform Sampling

**Figure 9:**
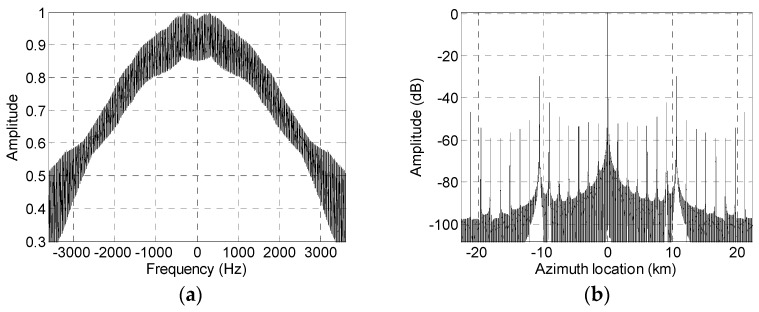

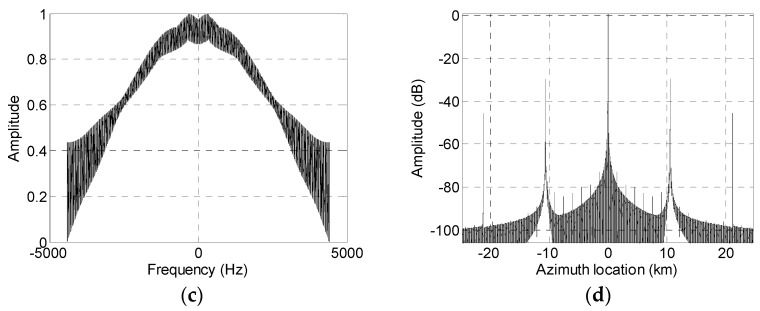
Reconstruction results of non-uniform sampling and adaptive phase center adjustment. (**a**) Reconstructed spectrum in the non-optimum PRF; (**b**) reconstructed pulse compression result in the non-optimum PRF; (**c**) reconstructed spectrum after adaptive phase center adjustment; and (**d**) reconstructed pulse compression result after adaptive phase center adjustment.

The seven-channel system was used in the simulation experiment. The optimum PRF of the system without adjusting receiving phase centers was 1234.3 Hz. Assuming that up to 30% of elements on each antenna was allowed to be turned off, the optimum value of the PRF could be adaptively adjusted within 1175.5–1299.2 Hz by invalidating elements at both ends of the antenna. Figure 9 gives the reconstruction result of a non-band-limited signal with 1300 Hz of the PRF in the case of conventional phase center spacing and adaptively adjusted phase center spacing. With respect to Figure 9a, false targets in Figure 9b were significantly reduced by phase center adaptive adjustment upon reception leaving only false targets caused by the non-band-limitation of the signal.

**Figure 10:**
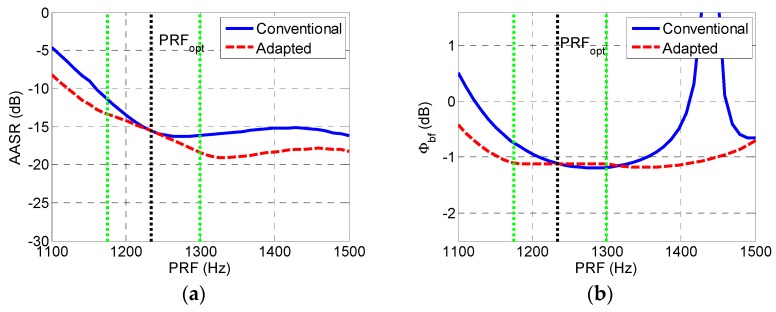
Simulated AASR and SNR scaling factor. (**a**) Simulated AASR and (**b**) simulated SNR scaling factor $\Phi_{\mathrm{bf}}.$

The corresponding AASR and SNR scaling factor $\Phi_{\mathrm{bf}}$ are shown in Figure 10. When the PRF value fell into the range 1175.5−1299.2 Hz, the AASR was consistent with the value of equivalent single channel SAR. For PRF values below 1175.5 Hz or higher than 1299.2 Hz, because of the non-uniform sampling, the AASR was higher than that of the equivalent monostatic signal but was obviously decreased with respect to the conventional multichannel reconstruction approach.

Regarding the SNR, when the PRF fell into 1175.5–1299.2Hz, the value of $\Phi_{\mathrm{bf}}$ remained constant independently of the PRF since the uniform sampling was ensured. For other values of the PRF, because of the non-uniform sampling, the AASR was higher than that in uniform sampling but was obviously lower than the conventional reference, as is shown in Figure 10b.

### 4.2. Effect of False Target Suppression

**Figure 11:**
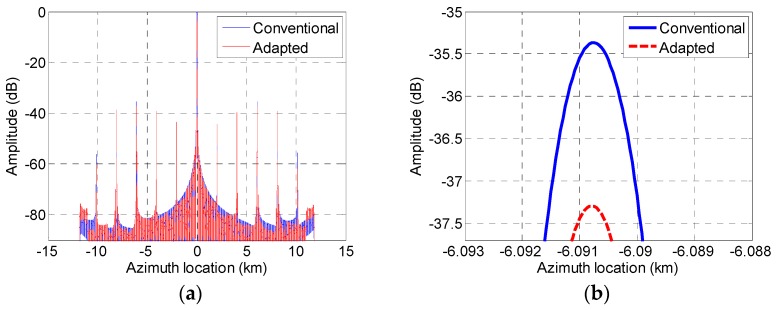
The effect of phase center adjusting on the suppression of false targets. (**a**) Reconstructed pulse compression results for uniform and non-uniform phase center and (**b**) the top of a fake target is zoomed in.

An example of phase center adjusting to suppress false targets is given in this section. The phase center positions before and after adjustment are listed in Table 2. Figure 11 verifies the suppression effect of phase center adaptively adjusting on the false targets. Figure 11a shows the reconstructed pulse compression results with the presence of channel errors; Figure 11b is an enlargement of Figure 11a at the top of a false target. It can be seen that false targets could be suppressed for about 2 dB after adaptive phase center adjustment. Though the suppression effect was not significant, the proposed method could further suppress the false targets after channel error compensation. This can also be regarded as an advantage of presented receiving phase center adjustment.

## 5. Conclusions

An advanced azimuth antenna architecture which allows for the adjustment of the phase center position of sub-apertures was proposed in this paper. Benefiting from receiving phase center adjustment, the performance of the HRWS SAR system can be improved in following three aspects. Firstly, the PRF of the system in a certain range can be regarded as the optimum value, so complex signal reconstruction algorithms will be omitted in novel systems. Secondly, non-uniform sampling that results in the severe degradation of imaging performance is avoided by adjusting the phase center if it falls into the optimum-PRF-range. Thirdly, by adaptively adjusting the phase center position of each channel, false targets caused by residual channel error after compensation can be further suppressed to some degree, and the quality of the resulting SAR image can be further improved.

In conclusion, receiving phase center adjustment is an effective method for compensating for non-uniform sampling and can suppress the false targets caused by channel error to a certain extent. However, since the number of elements that can be turned off cannot exceed a certain ratio, the optimum PRF can only be adjusted within a range, which restricts the usable PRF range of the system. In further research, the joint adjustment of transmit and receive phase centers will be considered to compensate for a wider range of the non-uniform PRF. Furthermore, the presented method can be extended to multiple-input multiple-output (MIMO) SAR systems to further improve the performance.

## Figures and Tables

**Table 1:** System simulation parameters.

Parameter (Azimuth)	Symbol	Value
Carrier frequency	$f_{c}$	9.6 GHz
Carrier wavelength	λ	0.031
Orbit height	$R_{0}$	895 km
Sensor velocity	$v_{s}$	7560 m/s
Overall Rx antenna length	$L_{a}$	12.25 m
Rx sub-aperture length	$l_{a}$	1.75 m
Elements number on each sub-aperture	K	60
Element size	*d*	0.03 m
Channels number	N	7
Desired PRF range	PRF	1100 Hz–1500 Hz

**Table 2:** Phase center position before and after adjusting.

Channels	1	2	3	4	5	6	7
$X_{n}$ before adjusting (m)	5.250	3.500	1.750	0	−1.750	−3.500	−5.250
$X_{n}$ after adjusting (m)	5.565	3.752	1.813	0	−1.855	−3.710	−5.502
